# Development of MEMS Multi-Mode Electrostatic Energy Harvester Based on the SOI Process

**DOI:** 10.3390/mi8020051

**Published:** 2017-02-13

**Authors:** Bongwon Jeong, Min-Ook Kim, Jae-Ik Lee, Youngkee Eun, Jungwook Choi, Jongbaeg Kim

**Affiliations:** 1 School of Mechanical Engineering, Yonsei University, 50 Yonsei-ro, Seoul 03722, Korea; jeongbw@gmail.com (B.J.); mokim@yonsei.ac.kr (M.-O.K.); sbrm@yonsei.ac.kr (J.-I.L.); yeun@yonsei.ac.kr (Y.E.); 2 School of Mechanical Engineering, Yeungnam University, 280 Daehak-ro, Gyeongsan 38541, Korea

**Keywords:** energy harvester, micro-electromechanical systems (MEMS), kinetic energy transduction, multi-mode system

## Abstract

Multi-vibrational-mode electrostatic energy harvesters are designed and micro-machined utilizing a simple silicon-on-insulator (SOI) wafer-based process. Enhanced adaptability to various vibrational environments is achieved in the proposed design by using serpentine springs attached to the fishbone-shaped inertial mass. The experimental results show that the developed device could convert an input vibration of 6 *g* at 1272 Hz to 2.96, 3.28, and 2.30 μW for different vibrational directions of 0°, 30°, and 45° with respect to a reference direction, respectively, when all serpentine springs are identical. An alternative device design using serpentine springs with different stiffnesses between *x*- and *y*-axes exhibited resonance frequencies at 1059 and 1635 Hz for an input vibrational direction of 45° and acceleration amplitude of 4 *g*, successfully generating 0.723 and 0.927 μW of electrical power at each resonance, respectively.

## 1. Introduction

The developments in micro/nanotechnology over the last few decades have promoted the birth of miniaturized devices with improved performance, most of which have replaced conventional macro-sized devices. Micro/nano-sized devices typically employ electrical energy for their operation, which is usually provided by an external energy source, such as a power supply or battery. However, conventional energy sources are usually large in volume and inevitably require periodic replacement, impeding further miniaturization and precluding quasi-permanent, independent operation of micro/nano-sized devices [1]. As an alternative to batteries, micro fuel cells have been proposed and have shown great performance in terms of miniaturization and the volume-to-power generation ratio. However, because micro fuel cells still require a periodic supply of fuel [2], the realization of self-sustained micro/nano-sized devices still remains challenging. 

As an alternative energy source, energy harvesting devices, which convert ambient energy into utilizable electrical energy, have been introduced. Natural energy sources for harvesters exist in many different forms, such as solar energy, kinetic energy, thermal energy, and radioactive energy. [3]. Among these energy sources, kinetic energy in the form of periodic vibration has received considerable attention, especially in micro-electromechanical systems (MEMS), for the use as a source of operation energy for self-sustained micro-sensors and communication nodes [4]. Vibration sources are easily found in common environments such as the human body, motor-driven machines, and home appliances, and they can be converted into electrical energy using simple MEMS structures [5]. The MEMS vibration energy harvesters are typically constructed as spring-mass systems with one or more resonance modes and frequencies of vibration. Depending on the transduction mechanism, the majority of MEMS vibration energy harvesters are categorized into three different types: piezoelectric, electromagnetic, and electrostatic harvesters [2]. While the piezoelectric and electromagnetic harvesters require the employment of complex fabrication steps with low process compatibility, electrostatic harvesters can be easily fabricated by standard micro-machining processes [2,3]. Electrostatic energy harvesters are a form of polarized capacitor with variable capacitance between fixed and movable electrodes; the stand-alone polarization is realized by an electret, a quasi-permanently charged material with a lifetime of a few decades [6].

In reality, vibration from various kinetic energy sources does not exhibit constant properties; the vibration may vary its direction during the operation and have a series of dominant frequencies. For example, the direction of vibration from home appliances is observed to be irregular in time [7]. To ensure optimal power generation, a vibrational energy harvester with a single degree of freedom (DOF) would not be sufficient in such conditions because the moving axis of the harvester should be well aligned with the direction of vibration in order to generate electrical energy. In addition, in terms of the frequency spectrum, a typical vibration source usually generates a series of dominant frequency peaks [8]. However, most previously reported kinetic energy harvesters are composed of a movable mass with a single DOF and a single resonant frequency [9,10,11], which results in a low energy conversion efficiency as a meaningful amount of energy can only be generated at the resonant frequency.

The conversion efficiency can be improved by designing a multi-mode spring-mass system such that the harvester can convert either multi-directional or multi-frequency vibration inputs to energy. Electret-based parallel-plate electrostatic harvesters have been suggested for multi-directional energy conversion [7,12]. These devices employ symmetrically-positioned circular-shaped springs attached to a centric mass in order to enable multi-directional displacement of the centric mass. However, the parallel-plate harvesters employ the change in linear capacitance for the energy conversion by changing the overlapping area between stacked plates. Such overlapping geometry intrinsically exhibits limited energy conversion efficiency compared to the gap-closing geometry with a nonlinear capacitance change, especially when an excessive input vibration magnitude is introduced to the system. Yang et al. have shown a new geometry based on radial comb-finger arrays with an overlapping mode for harvesting planar vibration [13]; this geometry is suitable for rotational vibration rather than multi-directional transverse vibration. On the other hand, a multi-directional electrostatic energy harvester based on the gap-closing mode of the comb-finger array has been reported by Zhu et al. [14], but the harvester is operated under ultrasonic vibrations that cannot be easily observed in the ambient environment.

In addition to electrostatic multi-directional harvesters, other studies have been conducted for converting diverse frequencies. An early approach integrated several piezoelectric cantilevers having different resonant frequencies onto a single device [4,15]. Roundy et al. suggested a piezoelectric energy harvester with multiple modes that generated a few resonant frequencies [16]. Multiple masses were connected to each other by mechanical flexures capable of generating the number of modes equivalent to the required number of the DOF. Yang et al. [17] have introduced a multi-mode electromagnetic harvester by employing the same approach suggested in [16]. Although the previous works have implemented multi-frequency energy harvesters well, each requires a rather complicated fabrication process due to the adopted materials or the structures. The piezoelectric harvester requires a series of fabrication processes to deposit and pattern a stack of thin films that avoidably leads to an increased cost of fabrication, whereas the electromagnetic harvester requires a coil structure and magnet-attaching process that is complicated to implement and inappropriate for mass-production.

In this work, we introduced multiple vibrational modes of an inertial mass for electrostatic energy harvesters that can convert multi-directional and multi-frequency vibrations to electric energy. The design employs comb-finger arrays as capacitive structures to enhance the capacitance variation even at small vibrations, and the device fabrication is easily implemented using a simple and parallel silicon-on-insulator (SOI) process. The employed serpentine springs enable movement of the inertial mass in both *x*- and *y*-axis, thus generating a mode for each axis. As experimentally shown in the following sections, the proposed device harvested energy with respect to multiple directions of vibration or at multiple frequencies, depending on the configuration of the spring sets. Our results demonstrate the potential for harvesting arbitrarily variable vibrations for utilizable electric energy.

## 2. Materials and Methods 

### 2.1. Design and Working Principle

**Figure 1:**
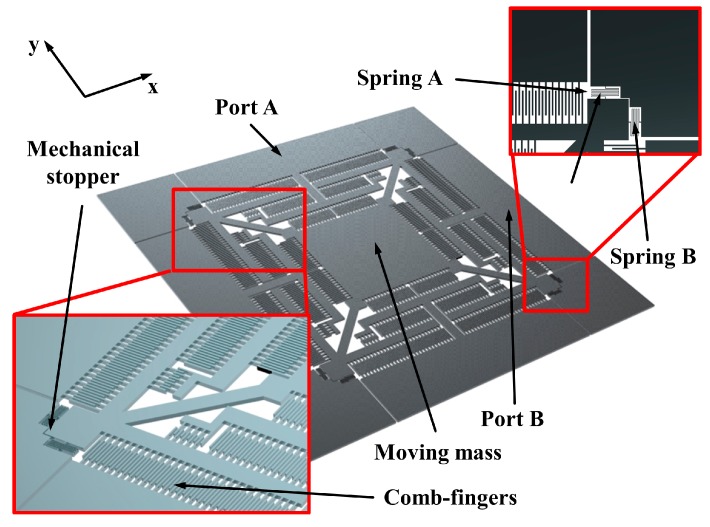
Schematic view of the electrostatic multi-mode energy harvester fabricated using a silicon-on-insulator (SOI) process.

Figure 1 shows the schematic view of the proposed electrostatic energy harvester with multiple vibrational modes. Unlike the conventional 1-DOF energy harvester, i.e., a simple beam structure such as a spring (and a mass), the presented device utilizes serpentine beams to enable inertia mass motion in both *x*- and *y*-axes. Eight serpentine springs (four springs for each set) are attached symmetrically to the inertial mass. The oscillation of the moving mass induces a capacitance change between the fixed and freely movable comb-finger arrays, which are attached to anchors and to the moving mass, respectively. To maximize the capacitance change in a given space for highly efficient energy conversion, comb-finger arrays with lengths of 450 and 250 µm are densely positioned on the fishbone-shaped inertial mass [18,19]. The initial gap between the comb-finger arrays is 11 µm, whereas its minimum gap during the oscillation is set at 1 µm, which is provided by mechanical stops at each edge of the inertial mass. The mechanical stops also keep both the free and fixed comb-finger arrays from becoming snapped in or electrically shorted. Assuming the ideal case without any parasitic capacitance, the initial capacitance is calculated to be 30.14 pF. When a 10-µm displacement is considered in both the *x*- and *y*-axes, the maximum capacitance in the ideal case is 173.7 pF. The detailed parameters of comb-fingers are given in Table 1. To minimize parasitic capacitance, which contributes to a deviation between the experimental and ideally calculated values, the backside (i.e., bottom-side) of the inertial mass and the comb-finger arrays is completely removed.

**Figure 2:**
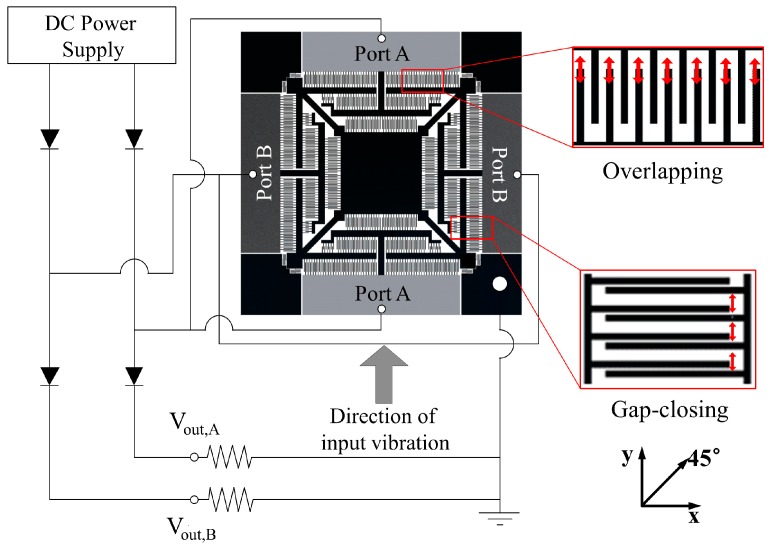
Working principle and power-processing circuit for the multi-mode energy harvester. The direct current (DC) power supply, assumed as an electret, provides the initial polarization of the energy harvester, whereas the integrated diodes allow the charge flow in a single direction. The displacement of the inertia by the external vibration induces a change in the capacitance, which in turn results in an increment of the polarized voltage in the energy harvester. Note that dominant energy conversion occurs at the port where the gap-closing behavior is observed because it significantly changes the total capacitance compared to the overlapping behavior.

The working principle and electric configuration of the energy harvester is depicted in Figure 2. The designed energy harvester is essentially a capacitor that requires polarization between the electrical ports to function as an energy transducer. It is common for the capacitive energy harvester to incorporate the polarization by integrating an electret to acquire the stand-alone harvesting functionality. It is noteworthy to mention that an electret is a quasi-permanent charged polymer, which serves as a reservoir of charges. Once an electret is attached to the conductive material, it can induce an electrical field inside the conductor by attracting charges from one point to the other, as of the relation between the magnet and the ferromagnetic material. An electret can be formed from numerous polymers including the photosensitive polymers, and the quasi-permanent encapsulation of charges is performed by exposing the polymer to the concentrated electric field generated by a voltage of a few thousands, which is called the corona-discharging process [6]. Instead of utilizing an electret itself, we have alternatively utilized a direct current (DC) power supply as a source of charges and assumed it as an integrated electret. A series of diodes have been utilized in the electrical circuit to prevent the backflow of charges to the power source when the polarized voltage in the energy harvester increases due to changing capacitance. The energy conversion occurs at the comb-finger arrays when the capacitance changes due to the displacement of the inertial mass by external vibration. Under the given direction of input vibration shown in Figure 2, the capacitance of the comb-finger arrays changes via the overlapping and gap-closing modes. The capacitance change via the gap-closing mode is significantly dominant compared to that via the overlapping mode as the displacement is only a few micrometer. In this regard, if we assume that the input vibration is given only along the *y*-axis and its frequency approaches the bandwidth of the energy harvester, the dominant energy harvesting occurs at port B. On the other hand, port A should dominate energy harvesting if the same condition is applied only along the *x*-axis.

The designed energy harvester has two discrete applications depending on the configuration of the serpentine springs. When springs A and B in Figure 1 are identical, the vibration modes corresponding to *x*- and *y*-axis will have the same resonant frequency, enabling multi-directional energy harvesting for a single resonance bandwidth. In the case where springs A and B are asymmetrically designed, the device will generate two modes with different resonant frequencies for each axis, enabling multi-frequency energy harvesting in a single direction (i.e., 45° from the reference frame). 

The resonant frequency for each axis in accordance to the spring configuration can be estimated by the following equation:

(1) $$f_{x}=\frac{1}{2\pi }\sqrt{\frac{k_{x}}{m}},\;f_{y}=\frac{1}{2\pi }\sqrt{\frac{k_{y}}{m}}$$

where subscripts denote the corresponding axis, *m* is the mass, and *f* and *k* are the resonant frequency and the effective stiffness, respectively. Considering that we have eight springs at each corner attached to the inertia in a symmetric manner, the effective stiffness can be expressed in the following form:

(2) $$k_{x}=4\left(k_{x.s1}+k_{y.s2}\right),\;k_{y}=4\left(k_{y.s1}+k_{x.s2}\right)$$

where *k_x.sn_* and *k_y.sn_* denote the stiffness of the *n*^th^ serpentine spring for *x*- and *y*-axis, respectively. The expressions for *k_x.sn_* and *k_y.sn_* of a general serpentine spring are given as [20]:

(3) $$k_{x.sn}=\frac{6EI_{z}}{\left(N+1\right)l_{0}^{3}}\;k_{y.sn}=\frac{3EI_{z}}{\left(8N^{3}+36N^{2}+55N+27\right)l_{p}^{2}l_{0}}$$

where *E* is Young’s modulus, *I_z_* is the moment of the inertia for the section of the spring, *N* is the number of folding, and *l*_p_ and *l*_o_ are the length of the segment beam parallel to *x*-axis and *y*-axis, respectively. It can be seen from Equations (2) and (3) that the effective stiffness will be identical (*k_x_* = *k_y_*) as long as the identical set of serpentine springs are utilized, thus resulting in the mode degeneracy (*f_x_* = *f_y_*). In an opposite point of view, the developed system will exhibit two different resonant frequencies for the *x*- and *y-*axis when two different sets of the serpentine springs are utilized (*k_x_* ≠ *k_y_*).

To verify the described operations, two electrostatic energy harvesters have been developed and examined: a multi-directional energy harvester with an identical set of 5-µm-wide serpentine springs, and a multi-frequency energy harvester with 5-µm-wide serpentine springs whose cross-section is facing *x*-axis (spring B) and the other springs with the width of 6 μm facing *y*-axis (spring A). The effective stiffness of the developed system and the corresponding resonant frequencies are calculated based on Equations (1)–(3), which are summarized in Table 2 and Table 3.

### 2.2. Fabrication

**Figure 3:**
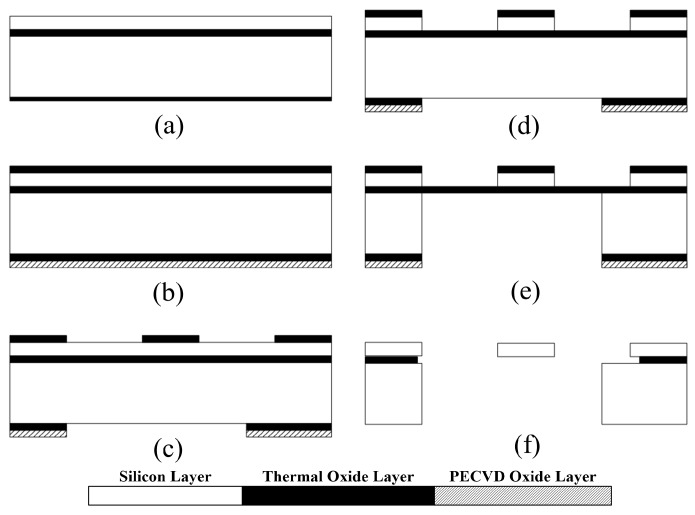
Fabrication process of the multi-mode energy harvester. (**a**) SOI wafer; (**b**) thermal oxide growth and plasma-enhanced chemical vapor deposition (PECVD) oxide deposition on the backside; (**c**) Photoresist patterning and oxide etching by reactive ion etching (RIE); (**d**) silicon device layer etching by deep reactive ion etching (DRIE); (**e**) substrate etching by DRIE; and (**f**) vapor phase HF etching of oxide layers.

**Figure 4:**
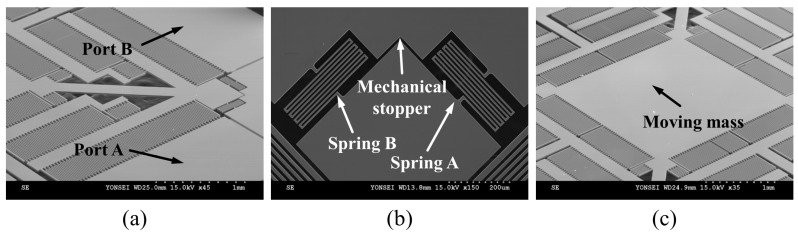
Scanning electron microscopy (SEM) images of the fabricated harvester. (**a**) Quadratic view showing comb-finger arrays, springs, and electrodes; (**b**) edge of the moving mass attached with spring A and B (identical design), whose excessive movement is restricted by a mechanical stopper; (**c**) wide view showing the moving mass and comb-finger arrays.

The device is fabricated on SOI wafer that consists of a device layer (50 µm), a buried oxide layer (2 µm), and a substrate (400 µm) as shown in Figure 3a. First, 1-µm-thick silicon dioxide layers are thermally grown on both sides of the wafer. Then, plasma-enhanced chemical vapor deposition (PECVD) is used to deposit an additional 3-µm-thick layer of silicon dioxide on the backside of the wafer to provide a sufficient mask for backside etching, as shown in Figure 3b. Photolithography is conducted to form masks for patterning the oxide layers on each side. To pattern the oxide layers, and reactive ion etching (RIE) is used to etch the silicon dioxide (Figure 3c). Provided with the silicon dioxide etch mask, deep reactive ion etching (DRIE) is conducted to form the designed structure on the device layer, as shown in Figure 3d. The device layer is then bonded upside down with the handle wafer using thermal grease, followed by the removal of the backside layer using DRIE. The handle wafer is used to secure etch uniformity by minimizing a leakage of cooling gas through the patterned front side during the backside DRIE. The dummy wafer is then detached from the processed SOI wafer by submerging the wafers in acetone, resulting in the structure of Figure 3e. The dummy wafer is then detached from the processed SOI wafer by submerging the wafers in acetone, resulting in the structure of Figure 3e. To avoid stiction, the devices are released under HF vapor [21], and the final structure is given in Figure 3f. Scanning electron microscopy (SEM, S-4300, Hitachi, Tokyo, Japan) images of the fabricated devices are shown in Figure 4.

## 3. Results and Discussion

**Figure 5:**
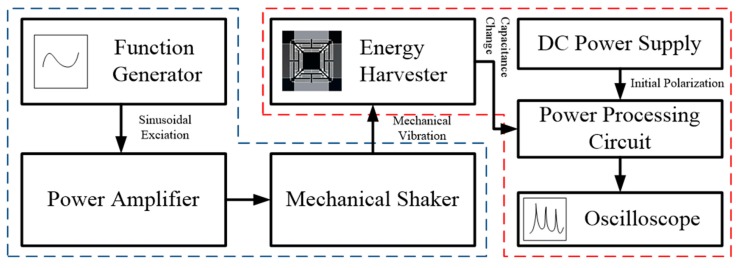
Experimental setup. The blue dotted line indicates the equipment setup for generating the mechanical vibration, whereas the red dotted line indicates the equipment setup for reading the generated voltage.

The schematic view of the experimental setup is given in Figure 5. The electrical signal for the input vibration is generated from a function generator and transferred to the power amplifier. The amplified signal is transmitted to the mechanical shaker where the energy harvester is mounted. The power processing circuit described in Figure 2 receives the initial polarization voltage from the DC power supply, which is then transferred to the connected energy harvester through the diodes. As the excitation vibration is exerted to the mounted energy harvester, the electrical signal, which indicates the harvested energy, is generated and measured by the oscilloscope connected to each electrical port of the load resistance in the power processing circuit. The diodes in this circuit should be carefully selected in order to harvest energy effectively because the excessive leakage current will hinder the energy harvesting process [22]. In this experiment, the switching diodes are FJH-1100 diodes by Fairchild Semiconductor (Sunnyvale, CA, USA).

**Figure 6:**
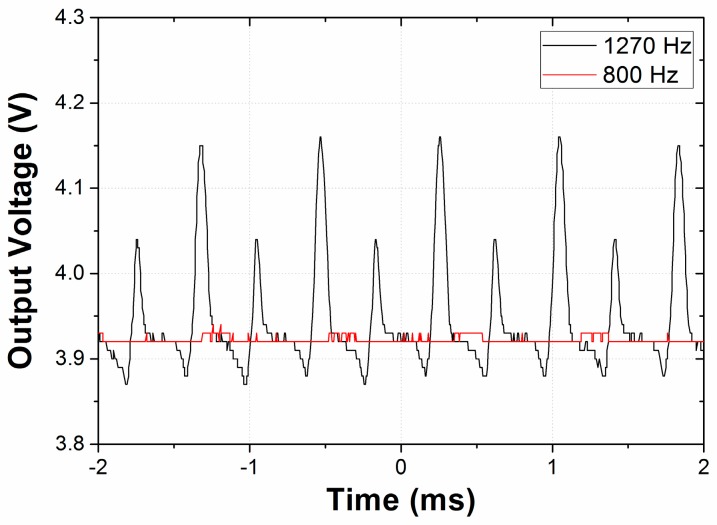
Output voltages from port B in the time domain. The multi-directional energy harvester (symmetrical spring design) is used under a vibration of 6 *g* with a tilting angle of 0° with respect to the *y*-axis, an initial polarization voltage of 5 V, and resistance of 0.717 MΩ. At the band of on-resonance (1270 Hz), a voltage peak indicating the energy conversion appears, whereas no significant change is shown at off-resonance (800 Hz.)

Figure 6 shows the comparison of output voltage between the cases when the input vibration is near or distant from the resonant frequency of the harvester. While no significant voltage oscillation is observed when the input vibration (800 Hz) is given at the frequency distant from the resonance, periodic oscillation of the output voltage is evidently observed in the case operated near the resonant frequency (1270 Hz). Two peaks exist in a single period of oscillation: a large peak corresponding to the inertia mass moving in a certain direction and a small peak corresponding to the mass moving in the opposite direction. The deviation in the amplitude between these two peaks may be caused by misalignment between the input vibration and the device orientation and/or asymmetry in the structure due to unavoidable fabrication error. To clearly show the dynamic behavior of the device near its resonance, we measured four consecutive energy conversion cycles, and the peak voltages are averaged to calculate the maximum (and peak) power for each frequency in the following spectra.

To evaluate the net maximum power generated solely by the energy harvester, the power from the polarization voltage source should be subtracted from the power observed during the energy conversion process. Thus, the following equation is used to evaluate the net maximum power of the energy harvester:

(4) $$P_{\max.\mathrm{net}}=\frac{V_{\max.\mathrm{vib}}^{2}}{R}-\frac{V_{\mathrm{no}.\mathrm{vib}}^{2}}{R}$$

where *P*_max.net_ is the net maximum power, *V*_max.vib_ is the average value of consecutive maximum voltage peaks for four cycles, and *V*_no.vib_ is the output voltage when no vibration is given. Due to power consumption by the diodes, *V*_no.vib_ is evaluated to be 3.92 and 8.84 V for the initial polarization voltage of 5 and 10 V, respectively. The net maximum power is determined by the resistance of the electrical load. For the case shown in Figure 6, where a resistance of 0.717 MΩ and polarization voltage of 5 V is employed at port B, the net maximum power is calculated as 2.59 µW.

### 3.1. Multi-Directional Energy Harvester

**Figure 7:**
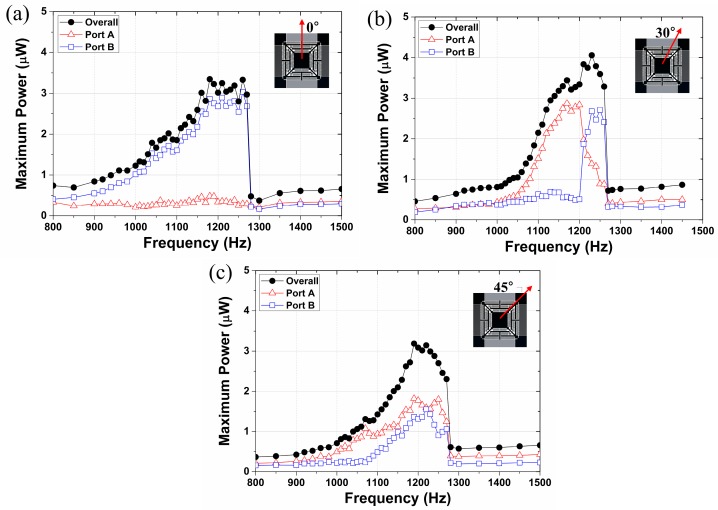
Experimental results for multi-directional energy harvester, exhibiting power generation at (**a**) 0°; (**b**) 30°; and (**c**) 90° input vibration.

The experiment on the multi-directional energy harvester is conducted by varying the direction of the input vibration at angles of 0°, 30°, and 45° with respect to *y*-axis. The initial polarization voltage is given as 5 V, the resistance is 0.717 MΩ, and the input acceleration is 6 *g*. The experimental result is depicted in Figure 7. The power generated at each port is combined to display the overall power generation, while the inset indicates the direction of the input vibration. The results show that the system has nonlinear dynamic behavior and its jumping frequency is observed as 1272 Hz, where the maximum power generated at the jumping frequency is evaluated as 2.96, 3.28, and 2.30 μW for a tilting angle of 0°, 30°, and 45°, respectively. The quality factor of the resulted dynamic response is estimated from Figure 4c, which was calculated as 14.6. As expected, port B (gap-closing mode) generates higher power than port A (overlapping mode) when the vibration is applied at 0° (Figure 3a). In Figure 3b, a slight deviation of the resonant peak from port A to port B can be observed, which is possibly due to the asymmetric electrostatic spring effect as the peak displacement of the inertia mass in the *x*- and *y*-axes differs.

### 3.2. Multi-Frequency Energy Harvester

**Figure 8:**
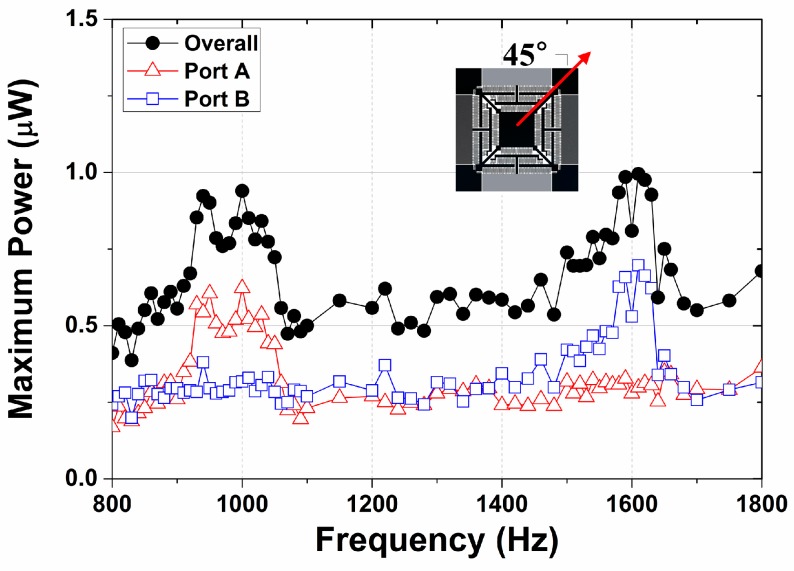
Experimental result for multi-frequency energy harvesting. The vibration is given at the tilting angle of 45° with an acceleration of 4 *g*. The result indicates that the resonant vibration occurs at 1059 and 1635 Hz in *x*-axis (port A) and *y*-axis (port B), respectively, and successfully generates electric power from the different resonant frequencies.

Figure 8 shows the experimental results for the multi-frequency energy harvester. An initial polarization voltage of 10 V is use, with a resistance of 0.717 MΩ and input acceleration of 4 *g*. The device is mounted with a tilting angle of 45° with respect to *y*-axis in order to exert the same amount of acceleration on both the *x*- and *y*-axes. The experimental results show that the jumping frequencies for port A and port B are observed as 1059 and 1635 Hz, respectively, whereas the maximum power at each frequency is 0.723 and 0.927 μW, respectively. Thus, kinetic energy can be converted from two discrete bandwidths even under single directional vibration by designing asymmetric springs. The device can clearly harvest ambient energy more effectively when the resonant frequencies of the system are designed to match the dominant frequencies of the environment.

### 3.3. Discussion

It is shown in Figure 7 and Figure 8 that the strong nonlinear dynamic response is extant, represented by the stiffness-hardening behavior and the bifurcation at the jumping frequency. It is noteworthy that the stiffness-softening behavior is usually expected in MEMS operating under the electric field, opposing the observation on the current result [23,24]. On the other hand, it is also reported that the oscillators whose both ends are constrained to the ground exhibit the stiffness-hardening dynamic response in the presence of the large deformation of the flexural component [25,26,27], also referred as geometric nonlinearity. We conjecture that the stiffness-hardening behavior arises from the geometrical configuration of the system and the excessive deformation of the serpentine spring that overcome the stiffness-softening behavior induced from the electrical field. Although the nonlinear resonance is quite difficult to be utilized for the energy harvesting in general, the observed nonlinear feature can be quite beneficial for the development of the energy harvester with the enhanced responsivity to the random vibration [25]; the existence of the stiffness-softening or -hardening behavior can extend the bandwidth of the dynamic system whose range depends on the amount of the input energy [26,27], and this broadened bandwidth can enhance the energy conversion efficiency in the environment with the random and excessive vibration. 

Table 4 shows the comparison of the developed device with the other electrostatic MEMS energy harvesters previously developed and studied. It presents various specifications, among which key parameters such as output power (μW), volumetric power density per acceleration (μW/cm^3^·g), and the capability to harvest random vibration are included. When purely accounting for the power density per acceleration, there are other devices with outstanding performance, and our device exhibits only a mediocre performance. However, when also considering the capability of harvesting the random vibration, our device exhibits a good power density per acceleration compared to the others. Regarding that there is still room for integrating an additional mass to the moving shuttle, an enhanced performance can be anticipated once further improvement is conducted through design optimization. 

## 4. Conclusions

In this study, a multi-mode electrostatic energy harvester is designed, fabricated, and verified. Serpentine springs enabled bi-directional motion of the inertial mass, allowing the whole system to be operable at multiple vibrational modes. The capacitance change is maximized by adopting fishbone-like comb-finger arrays and by the gap-closing mode operation. Depending on the symmetry of the sets of serpentine springs, the developed energy harvester can be used for two discrete multi-mode environments. The experimental results validate that the device could convert vibrational energy with continuously varying characteristics, namely random directional movement or multi-frequency environments, into electrical energy.

## Figures and Tables

**Table 1:** Parameters for the comb-finger arrays in the device.

Comb Length	Gap	Initial Overlapping Length	Thickness	Total No. of Pairs	Effective No. of Pairs for a Single Axis
450 µm	11 µm	400 µm	50 µm	888	444
250 µm	11 µm	200 µm	50 µm	96	48

**Table 2:** Parameters for each type of serpentine spring.

Type	N	*w*	*l_o_*	*l_p_*	*I*_z_	*k_x.sn_*	*k_y.sn_*
Spring A	3	5 µm	325 µm	10.5 µm	5.21 × 10^−22^ m^4^	3.85 N/m	10.1 N/m
Spring B	3	6 µm	325 µm	9.5 µm	9.0 × 10^−22^ m^4^	6.65 N/m	21.3 N/m

**Table 3:** Parameters for each type of developed energy harvesters.

Type	*m*	*k_x_*	*k_y_*	*f_x_*	*f_y_*
Multi-directional (Spring A only)	1.45 × 10^−6^ kg	55.66 N/m	55.66 N/m	986.0 Hz	986.0 Hz
Multi-frequency (Spring A and B)	1.45 × 10^−6^ kg	66.85 N/m	100.4 N/m	1081 Hz	1324 Hz

**Table 4:** Comparison with the other capacitive energy harvesters.

Ref. No.	Power (μW)	Frequency (Hz)	Input Acceleration (*g*)	Volume (cm^3^)	Power Density Per Acceleration (μW/cm^3^·g)	Capability to Harvest Random Vibration
Our work	3.28	1272	6	0.0041	133.3	O
Arakawa [28]	6	10	0.4	0.8	18.75	X
Despesse [29]	70	50	0.8	0.0324	2700	X
Sheu [11]	0.0924	105	0.1	0.0045	205	X
Yang [13]	0.35	110	2.5	0.0394	3.55	O
Jia [30]	0.166	200	0.5	0.000278	1200	O
Tao [31]	0.0048	66	0.05	0.0588	1.64	O
Chiu [32]	1.2	1870	3.3	0.6	0.606	X
Basset [33]	0.061	250	0.25	0.06149	3.96	X
